# Bonding Performance of a New Resin Core System with a Low-Polymerization-Shrinkage Monomer to Root Canal Dentin

**DOI:** 10.3390/polym16233389

**Published:** 2024-11-30

**Authors:** Takashi Hatayama, Tomoko Tabata, Kota Kibe, Masaomi Ikeda, Yasunori Sumi, Yasushi Shimada

**Affiliations:** 1 Department of Cariology and Operative Dentistry, Division of Oral Health Sciences, Graduate School of Medical and Dental Sciences, Institute of Science Tokyo, Tokyo 113-8549, Japan; tabope@tmd.ac.jp (T.T.); kotakibe6226@icloud.com (K.K.); lucanus93@gmail.com (Y.S.); shimada.ope@tmd.ac.jp (Y.S.); 2 Department of Oral Biomedical Engineering, Graduate School of Medical and Dental Sciences, Institute of Science Tokyo, Tokyo 113-8549, Japan

**Keywords:** resin core build-ups, endodontically treated tooth, low polymerization shrinkage, root dentin, microtensile bond strength, bulk fill, SS-OCT, gap analysis

## Abstract

Resin core build-ups following root canal treatment still have many issues. This study evaluated whether a new low-polymerization-shrinkage resin core system (LC2) could address these issues by assessing its bonding performance to root canal dentin using microtensile bond strength tests and gap formation using swept-source optical coherence tomography (SS-OCT). Twenty-four extracted human lower premolars were used for bonding performance tests, while forty-eight sound extracted human wisdom teeth were used for gap observation. Four systems were compared: Luminous Core LC flow (LC1), LC2, MI Core LC flow (MIC), and Filtek Fill & Core (FFC). Cylindrical cavities were prepared, bonded, and filled with resin cores, and gap formation was evaluated. The results showed significant differences in bond strength between the coronal and apical sides: LC1 (coronal: 29.9 ± 3.8 MPa; apical: 12.4 ± 2.0 MPa), LC2 (coronal: 31.2 ± 3.6 MPa; apical: 17.8 ± 3.6 MPa), MIC (coronal: 28.7 ± 3.8 MPa; apical: 8.8 ± 2.1 MPa), and FFC (coronal: 29.0 ± 4.2 MPa; apical: 9.5 ± 1.9 MPa). LC2 exhibited significantly higher bond strength at the apical side compared to the other systems (*p* < 0.05). Gap formation was significantly reduced in LC2 (10.9 ± 5.0%) and FFC (11.9 ± 5.0%) compared to LC1 (31.8 ± 10.5%) and MIC (32.0 ± 5.6%) (*p* < 0.05). These findings suggest that LC2 is advantageous for resin core build-ups, particularly in improving adhesion to root canal dentin and reducing gap formation.

## 1. Introduction

In recent years, there has been a significant increase in the use of direct resin core build-ups in endodontically treated teeth [1]. The main reasons for this are the improved mechanical properties of the resin core itself compared to the past, the higher bond strength to dentin achieved by direct methods compared to indirect methods, and the contribution to reducing coronal leakage associated with these factors. Improving the seal at the interface between the restorative material and tooth structure is crucial in preventing the ingress of oral fluids and bacteria, which can compromise the longevity of the restoration [2,3]. However, challenges such as difficulty in removing moisture in the apical region and solvent removal from adhesives remain, which could jeopardize the long-term success of restorations. Therefore, various methods have been devised to solve these problems, but it is often reported that the adhesive performance to apical root canal dentin significantly decreases compared to the coronal side [4,5,6,7,8].

Similarly to adhesive strength, marginal adaptation is a key factor for achieving positive clinical results [9,10]. Due to the high C-factor within the root canal, polymerization shrinkage stress becomes significant, potentially compromising the seal at the dentin–adhesive interface and leading to gap formation [11]. Consequently, marginal adaptation, which correlates with adhesive strength, and adequate polymerization of the adhesive are essential during resin core build-up.

The development and evaluation of new resin core systems with low polymerization shrinkage could revolutionize resin core build-ups by minimizing issues like coronal leakage, which is crucial for the prognosis of endodontically treated teeth. Previous studies have highlighted the significant impact of coronal leakage in endodontically treated teeth, emphasizing the need for improved adhesive technologies that can securely seal the bonding interface [9,10].

While there are various methods for evaluating restorations, swept-source optical coherence tomography (SS-OCT), a non-destructive technique that does not use X-rays, provides high-resolution cross-sectional images at the micron scale of internal structures [12] and has been applied in dentistry for characterizing caries [13], evaluating gaps at the interface of composite materials and teeth in 2D and 3D images [14,15], and assessing internal gaps and defects in restorations [16]. Researchers have used SS-OCT to assess the marginal fit level of restorations [14,17,18,19], and some have evaluated the cavity adaptation of restorations using SS-OCT to study its correlation with confocal laser scanning microscopy (CLSM) [20] and scanning electron microscopy (SEM) [21].

This study aimed to examine whether a new low-polymerization-shrinkage (LPS) resin core system, developed with LPS monomers initially created for bulk-fill resin composites (SUN MEDICAL Co., Ltd, Shiga, Japan), could address the various challenges encountered in resin core build-ups. LPS monomers, first introduced as innovative components in dental restorative materials, are designed to reduce polymerization shrinkage through steric bulky groups and urethane moieties. Their low-polymerization-shrinkage performance and superior depth of cure have been well demonstrated in bulk base materials, making them highly suitable for clinical applications [22,23].

This research aimed to evaluate the adhesive performance to dentin and analyze adhesive interface gaps using swept-source optical coherence tomography (SS-OCT). Specifically, this study sought to enhance bond strength in the coronal and apical portions of root canal dentin, which are typically prone to inadequate adhesion due to anatomical and material challenges. By leveraging the unique properties of LPS monomers, including their ability to lower shrinkage stress without significantly compromising mechanical strength, this study aims to address the shortcomings of current resin core materials and contribute to more reliable and durable restorative outcomes. The null hypothesis of this study is that there are no significant differences in bonding performance and gap formation between the new low-polymerization-shrinkage resin core system and conventional systems when applied to root canal dentin.

## 2. Materials and Methods

### 2.1. Specimen Preparation for μTBS Test

Twenty-four caries-free, single-rooted human mandibular premolars with consistent root lengths were selected following ethical approval by the Ethics Committee of Tokyo Medical and Dental University, under protocol 2013022. The sample size was determined based on a pilot study, which estimated the mean bond strength of the experimental group to be approximately 30 MPa and that of the control group to be about 15 MPa, with standard deviations of ±4 MPa and ±3 MPa, respectively. A *t*-test with Bonferroni correction was assumed for the statistical method, and a total sample size of *n* = 20 was established using a power analysis software. Due to several pretest failures, an additional four samples were included, resulting in a total of *n* = 24 per group. These teeth were stored at 4 °C in distilled water and used within six months post-extraction. The crown portions were sectioned just below the cement–enamel junction using a precision diamond saw (Isomet, Buehler, Lake Bluff, IL, USA). Endodontic files were used for canal preparation, and post spaces (8 mm depth and 1.5 mm diameter) were created using FibreKor drills (Pentron, Wallingford, CT, USA) in a low-speed handpiece with copious water cooling. Post cavities were thoroughly rinsed with distilled water and dried using paper points. To support microtensile bond strength tests and prevent light from affecting the curing process through the thin dentin walls, the external surfaces of the roots were covered with Clearfil AP-X resin composite (Kuraray Noritake Dental, Tokyo, Japan). 

### 2.2. Bonding Procedure

Resin core build-ups were performed using four systems: Luminous Core LC flow (LC1) with Luminous bond (SUN MEDICAL Co., Ltd.), a new low-polymerization-shrinkage resin core DP-031 (LC2) with DP-032 Bond (SUN MEDICAL Co., Ltd.), MI Core LC flow (MIC) with G-Premio BOND (GC Corporation, Tokyo, Japan), and Filtek Fill & Core (FFC) with Scotchbond Universal Plus Adhesive (3M Company, Maplewood, MN, USA). The materials used in this study, along with their compositions, application methods, and mechanical properties, are summarized in Table 1 and Table 2. The curing modes for 1-SEA were dual-cure for Luminous bond and DP-032 Bond, and light-curing for G-Premio BOND and Scotchbond Universal Plus Adhesive. The application procedure for each system followed the manufacturer’s instructions. For resin core construction, light-curing was carried out according to each manufacturer’s specified duration (Valo LED Curing Light, high-power mode, 1400 mW/cm^2^, Ultradent, South Jordan, UT, USA). All procedures were performed under constant temperature and humidity conditions at 23 ± 1 °C and 60% relative humidity.

### 2.3. Microtensile Bond Strength (μTBS) Testing

**Figure 1:**
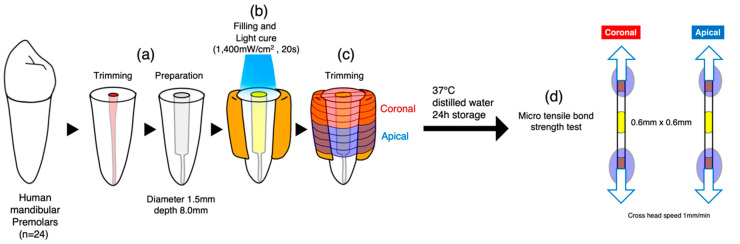
Specimen preparation: (**a**) The crowns of 24 lower premolars were removed and post cavities were prepared. (**b**) The external surfaces were built up with resin composite. The cavity was dried with paper points prior to the application of adhesives. The application procedure for each system followed the manufacturer’s instructions. For resin core construction, light-curing was carried out according to each manufacturer’s specified duration. (**c**) The specimens were sectioned into 8 slabs (4 coronal, 4 apical). (**d**) The slabs were sectioned into 0.6~0.6 mm^2^ stick-shaped beams. µTBS was performed.

After storage at 37 °C for 24 h, the specimens were sectioned into eight slabs perpendicular to the adhesive interface using a low-speed diamond saw under water cooling. Each tooth’s slabs were divided into two subgroups: coronal and apical, each 4 mm in length. The slabs were sectioned into 0.6 × 0.6 mm^2^ stick-shaped beams, and the dimensions of the cross-sectional area were checked with a digital caliper (Mitutoyo CD15, Mitutoyo, Kawasaki, Japan). The beams were attached to the testing jig using cyanoacrylate adhesive (Zapit, DVA, Anaheim, CA, USA), and a tensile load was applied using a tabletop testing machine (EZ Test Shimadzu, Kyoto, Japan) at a crosshead speed of 1 mm/min until fracture occurred (Figure 1). The force at fracture was recorded in Newtons (N) and converted to μTBS values (MPa). Since Levene’s test confirmed homogeneity of variances across groups, the μTBS data were analyzed using two-way ANOVA and *t*-tests adjusted to a 5% significance level by the Bonferroni method.

### 2.4. Failure Mode Analysis

Both the resin and dentin surfaces of the fractured beams were dehydrated and then mounted on brass stubs. Subsequently, they were sputter-coated with gold to facilitate conductivity for scanning electron microscopy (SEM). The fracture surfaces were examined using SEM (JSM-IT100, JEOL, Tokyo, Japan) to determine the mode of failure. Four distinct failure modes were classified: cohesive failure in the core material (characterized by failure predominantly within the core material, exceeding 70% of the area), adhesive failure (identified by failure primarily within the adhesive resin or at the resin–dentin interface, exceeding 70% of the area), cohesive failure in dentin (indicated by failure mainly within the dentin, exceeding 70% of the area), and mixed failure (a combination of cohesive and adhesive failures).

### 2.5. Specimen Preparation for Gap Analysis

**Figure 2:**
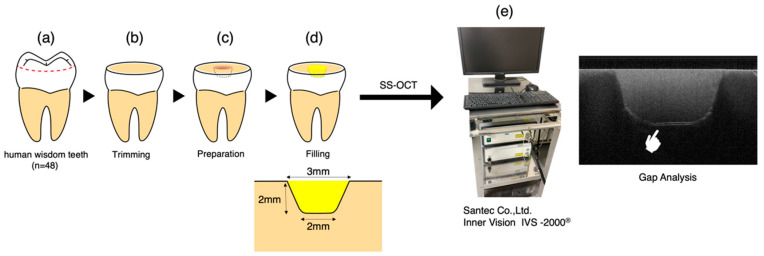
Specimen preparation for gap analysis: (**a**) Forty-eight sound extracted human wisdom teeth were selected. (**b**) After sectioning the occlusal enamel with a precision diamond saw, the surface layer was polished with 800-grit SiC paper to create a flat dentin surface. (**c**) Tapered cylindrical cavities (upper diameter: 3 mm, lower diameter: 2 mm, depth: 2 mm, no pulp exposure) were prepared using a high-speed handpiece with a diamond bur under water cooling. The samples were randomly divided into four groups (12 samples each) according to the materials used. (**d**) The cavities were treated following the manufacturers’ instructions for adhesive application and resin core build-up procedures. (**e**) Samples were observed using SS-OCT, and 2D images were captured. Each sample was fixed on the precise head stage of the SS-OCT, and a scanning laser beam was directed perpendicularly onto the restoration surface.

The procedural schematic is presented in Figure 2. For the gap analysis, forty-eight sound extracted human wisdom teeth were selected. After sectioning the occlusal enamel with a precision diamond saw (Isomet, Buehler, Lake Bluff, IL, USA), the surface layer was polished with 800-grit SiC paper (Sankyo, Saitama, Japan) to create a flat dentin surface. Subsequently, tapered cylindrical cavities (upper diameter: 3 mm, lower diameter: 2 mm, depth: 2 mm, no pulp exposure) were prepared using a high-speed handpiece with a diamond bur (SF207CR; Shofu, Kyoto, Japan) under water cooling. Procedures of specimen preparation and OCT real-time monitoring followed a previous study [24]. The samples were randomly divided into four groups (12 samples each) according to the materials used. The cavities were treated following the manufacturers’ instructions for adhesive application and resin core build-up procedures.

### 2.6. Gap Analysis Using SS-OCT

The SS-OCT system used in this study (IVS-2000, Santec Corporation, Komaki, Japan) utilizes a swept-source mechanism. It employs a high-speed laser that scans the 1260–1360 nm range (with a central wavelength of 1310 nm) at a sweep rate of 20 kHz. The system offers high resolution, with an axial resolution of 11 μm in air, corresponding to approximately 7 μm in dental tissues with a refractive index of about 1.5 [25]. Its lateral resolution is about 17 μm, determined by the objective lens of the probe. The laser output from the probe is 5 mW, which is within the safety standards of the American National Standards Institute.

The cavity adaptation of adhesive systems was assessed non-destructively and directly using this system. Based on a Michelson interferometer configuration, the probe scans the sample at designated X and Z positions, capturing backscattered light. This information is digitized in the time domain and transformed into depth information (A-scan) via Fourier analysis. A series of A-scans produces a cross-sectional image (B-scan), which is ultimately visualized as a grayscale image with 2001 × 1019 pixel resolution. This methodology enables direct assessment of the cavity adaptation and gap analysis of each adhesive system.

Samples were observed using SS-OCT, and 2D images were captured. Each sample was fixed on the precise head stage of the SS-OCT, and a scanning laser beam was directed perpendicularly onto the restoration surface. The samples were moved across the laser beam to capture cross-sectional B-scan images at maximum depth. If there were gaps at the interface between the restoration and the tooth, light partially reflected between the media of different refractive indices would appear as bright regions on the OCT images.

**Figure 3:**
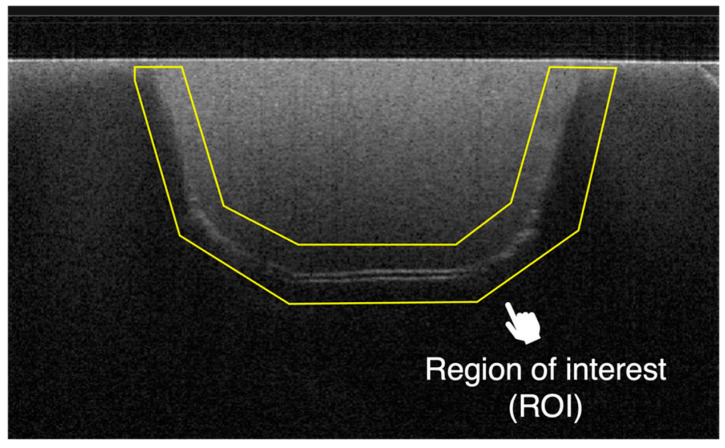
Location of the region of interest in the SS-OCT image. The proportion of white pixels (indicating gaps) within the ROI was totaled to calculate the sealed interface percentage (SI%).

In this study, the adhesive performance was quantitatively assessed using the sealed interface percentage (SI%) as an indicator. Two-dimensional SS-OCT images were imported into ImageJ software (version 1.53) and processed with a median filter to decrease background noise and enhance image quality [26]. An experimental threshold determination algorithm developed as an ImageJ plugin was used for image analysis. The region of interest (ROI) was selected as a polygon covering the entire length of the restoration interface (Figure 3). The proportion of white pixels (indicating gaps) within the ROI was totaled to calculate the SI%.

The data underwent normality testing and appropriate statistical tests were selected. The mean values of SI% were statistically analyzed using the Kruskal–Wallis test for multiple comparisons, and all analyses were conducted using SPSS.

## 3. Results

### 3.1. Results of Microtensile Bond Strength (μTBS) Testing

**Figure 4:**
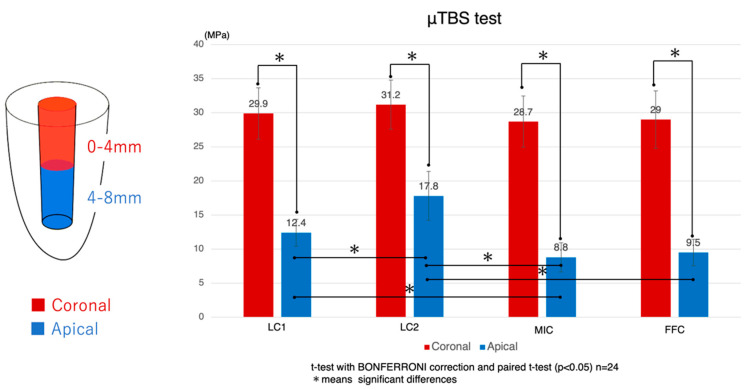
Microtensile bond strength to root canal dentin: mean ± S.D. (MPa): Bar with an asterisk indicates significant differences between groups (*p* < 0.05). The red bars represent the coronal side, while the blue bars indicate the apical side. LC1: Luminous Core LC flow (SUN MEDICAL Co., Ltd.); LC2: a new low-polymerization-shrinkage resin core DP-031 (SUN MEDICAL Co., Ltd.); MIC: MI Core LC flow (GC Corporation); FFC: Filtek Fill & Core (3M Company).

The evaluation of microtensile bond strength (μTBS) has provided new insights into the performance of a new low-polymerization-shrinkage resin core system (LC2). The analysis initially focused on comparing the bond strengths to root canal dentin at the coronal and apical parts of the teeth (Figure 4).

μTBS values were recorded for four different resin core systems labeled LC1, LC2, MIC, and FFC. Specifically, the μTBS values were as follows: for the LC1 group, coronal side: 29.9 ± 3.8 MPa, apical side: 12.4 ± 2.0 MPa; for the LC2 group, coronal side: 31.2 ± 3.6 MPa, apical side: 17.8 ± 3.6 MPa; for the MIC group, coronal side: 28.7 ± 3.8 MPa, apical side: 8.8 ± 2.1 MPa; and for the FFC group, coronal side: 29.0 ± 4.2 MPa, apical side: 9.5 ± 1.9 MPa. In all systems, the bond strength at the apical side was statistically significantly lower than at the coronal side. Statistical analysis was conducted using a *t*-test with Bonferroni correction and a paired *t*-test, and the differences in adhesive strength between the coronal and apical sides were statistically significant, with *p*-values < 0.05.

Focusing next on the adhesive performance exclusively at the coronal root canal dentin, no statistically significant differences were observed between the various resin core systems. However, when examining the apical root canal dentin, the LC2 group demonstrated significantly higher values compared to the other groups. Additionally, the LC1 group showed significantly higher values when compared to the MIC group, but no significant difference was observed compared to the FFC group. The MIC group exhibited significantly lower values than both the LC1 and LC2 groups, but no significant difference was found when compared to the FFC group. The FFC group, on the other hand, showed significantly lower values compared to the LC2 group, while no significant differences were noted when compared to the LC1 and MIC groups.

### 3.2. Results of Failure Mode Analysis

**Figure 5:**
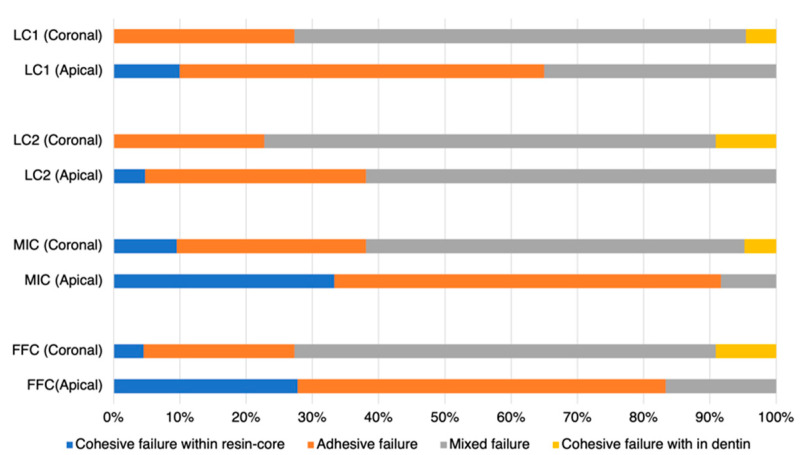
Failure mode distributions: cohesive failure in the core material (characterized by failure predominantly within the core material, exceeding 70% of the area), adhesive failure (identified by failure primarily within the adhesive resin or at the resin–dentin interface, exceeding 70% of the area), cohesive failure in dentin (indicated by failure mainly within the dentin, exceeding 70% of the area), and mixed failure (a combination of cohesive and adhesive failures).

Upon observing the failure mode results, it was noted that the coronal side exhibited more mixed failures compared to the apical side (Figure 5). Examining the failure modes at the apical side, the LC1 group showed a predominance of adhesive failures, while the LC2 group displayed a higher incidence of mixed failures. Both groups had fewer instances of cohesive failures within the resin core. However, the MIC and FFC groups experienced a higher proportion of both cohesive failures within the resin core and adhesive failures.

### 3.3. Results of Gap Analysis Using SS-OCT

**Figure 6:**
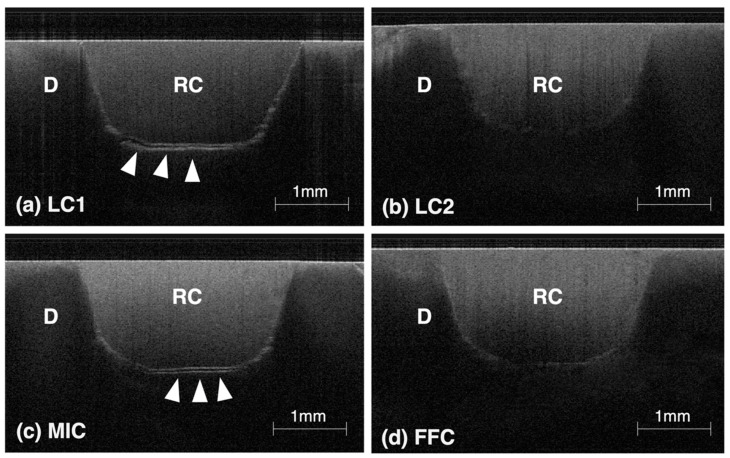
Representative SS-OCT images for evaluating marginal adaptation: In these images, “RC” denotes the resin core material, and “D” stands for dentin. Interface delamination and voids manifest as strong signals, visible as white lines. In groups LC1 (**a**) and MIC (**c**), many samples displayed pronounced gap formation (indicated by white arrows). Conversely, while the LC2 (**b**) and FFC (**d**) groups demonstrated regions of increased brightness, no samples showed distinct signs of delamination.

**Figure 7:**
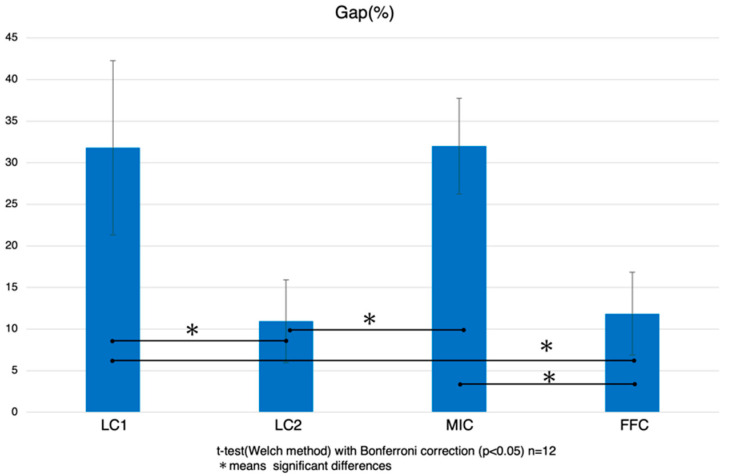
Gap analysis using SS-OCT: Using ImageJ software (version 1.53), gaps were measured and subsequently graphed. A bar with an asterisk indicates significant differences between groups (*p* < 0.05).

The results of the gap analysis derived from SS-OCT are presented in Figure 6 and Figure 7. This non-destructive imaging technique provided critical insights into the quality of the adhesive interface, particularly focusing on gaps that could compromise the long-term integrity of dental restorations. The diagnostic images revealed varying degrees of gap formation across different groups. Notably, the LC2 system exhibited the lowest percentage of gaps, with an average gap size of 10.9 ± 5.0%. It was followed by the FFC group with 11.9 ± 5.0%, then the LC1 group at 31.8 ± 10.5%, and the MIC group showed the highest incidence of gaps at 32.0 ± 5.6%. Statistically, the LC2 group had significantly fewer gaps compared to the LC1 and MIC groups, with no significant difference from the FFC group. Similarly, the FFC group also had significantly fewer gaps compared to the LC1 and MIC groups, while no significant difference was observed between the LC1 and MIC groups.

The results indicate that the LC2 and FFC groups show the best marginal adaptation to dentin cavities at a depth of 2 mm, demonstrating effective sealing properties. 

## 4. Discussion

Adhesion to root canal dentin near the apex is extremely challenging [3,4,5]. In this study, the new low-polymerization-shrinkage resin core group, LC2, exhibited significantly higher bond strength compared to other systems. This is clinically very useful and holds potential for enhancing core system stability, preventing microleakage, and avoiding fractures by integrating with the tooth root. Therefore, the null hypothesis that no significant differences exist in bonding performance and gap formation between the LC2 resin core system and conventional systems was rejected. This result highlights the superior adhesive performance and reduced gap formation achieved by the LC2 system.

First, the reasons why the LC2 system showed significantly higher bond strength to the apical root canal dentin compared to the LC1 system are discussed. Compared to the conventional LC1, the LC2 uses a resin core with similar composition and curing depth, which suggests that the significant difference observed is primarily due to reduced polymerization shrinkage. This is supported by SS-OCT observations showing significant differences in gap formation. Previous studies have reported that bulk-fill systems show less gap formation observed via SS-OCT compared to conventional shrinkage resin [27].

Next, regarding the significant difference observed between the LC2 group and the MIC and FFC groups, the use of light-cured bonds in the MIC and FFC groups and the incomplete curing towards the apex may be factors. Additionally, the curing depths of the resin cores for each group were shallower at 3.4 mm and 4.3 mm compared to LC1 and LC2, suggesting that the resin core might not have sufficiently polymerized near the apex. Furthermore, the depth of light-curing of the resin core is crucial as it facilitates curing of the unbonded top layer of the bonding material [28]. If the system does not promote polymerization through contact between the resin core and bonding material, then light passing through the resin core can finally cure the unbonded layer on the surface of the bonding material. Observations of the fracture morphology also suggest that both systems are likely experiencing curing deficiencies, as coalescence fractures and interfacial fractures are frequently observed.

In the SS-OCT cavity adaptation results, the LC2 and FFC groups showed significantly fewer gaps. Comparing the LC1 and LC2 groups, the use of a similarly composed bond and the fact that the 2 mm depth of the cavity allows sufficient light penetration suggest that the low polymerization shrinkage of the LC2 resin core contributed to this difference. While the LC2 bond has improved chemical polymerization properties compared to that of LC1, the 2 mm depth of the cavity ensures that the difference is minimally impactful because the light sufficiently penetrates this depth. The FFC also has low-polymerization-shrinkage properties, which is considered a reason for the reduced gap formation. However, even with bulk-fill materials, past studies have suggested that achieving 0% gap formation is not possible [27,29], and incremental filling is recommended when the cavity space is wide. However, in cases where a fiber post is inserted, incremental filling cannot be performed within the root canal; thus, a system like LC2 that results in fewer gaps and significantly higher adhesion up to the apex is considered more useful. 

These findings have significant clinical implications. The LC2 system, with its superior bond strength and reduced gap formation, addresses critical challenges in resin core build-ups, particularly in endodontically treated teeth. Its enhanced adhesion at the apical region mitigates common issues such as microleakage and secondary caries, which are often observed in traditional materials. Moreover, the minimized polymerization shrinkage and improved curing depth of LC2 suggest the potential for better marginal adaptation, even in anatomically challenging cases. These advantages may contribute to the durability of restorations and offer clinicians a more reliable material for achieving long-term success in dental restorations. 

Although this study demonstrated that the LC2 system significantly improved bond strength to apical root canal dentin and reduced gap formation compared to conventional systems, several limitations must be acknowledged. First, this study was conducted in vitro, which may not fully replicate clinical conditions. In vivo factors such as moisture, temperature fluctuations, and anatomical variations could influence adhesive performance. Second, the long-term durability of the adhesive interface was not evaluated in this study. While fewer initial interfacial gaps have been associated with better maintenance of bond strength after thermal cycling [24], further investigations incorporating thermal cycling or fatigue testing are required to assess the long-term clinical performance of the LC2 system. Finally, variations in tooth structure and preparation methods might affect the generalizability of the results. These limitations highlight the need for additional research to validate the clinical relevance and durability of the LC2 system in a broader range of scenarios.

## 5. Conclusions

This study demonstrated that the new low-polymerization-shrinkage resin core system (LC2) significantly improved bond strength to apical root canal dentin and reduced gap formation compared to conventional systems. These findings suggest that LC2 addresses critical challenges in resin core build-ups, particularly in endodontically treated teeth, by enhancing adhesion, minimizing microleakage, and potentially improving the long-term stability of restorations. However, bonding to apical root canal dentin remains a challenging issue, and further studies are required to address these difficulties and enhance the clinical performance of resin core systems.

## Figures and Tables

**Table 1:** Overview of materials, compositions, and application protocols used in this study.

Resin	Bonding	Compositions	Protocol	Code
Luminous core LC flow (Light cure)	Luminous bond (Dual-cure)	[Resin] Barium silicate glass, Methacrylic esters (Bis-MPEPP, aromatic diol-based methacrylic esters, others), and others. [Bonding] Methacrylic esters (4-META, others), acetone, water, and others (Brush: aromatic amines, aromatic sulfinate salts).	1. Mix the bonding with brush for 5 s and apply to the cavity. 2. After 20 s, air-blow 5 to 10 s. 3. Light-cure for 10 s. 4. Fill the resin core material. 5. Light-cure for 20 s.	**LC1**
DP-031 Resin core (Light cure)	DP-032 Bond (Dual-cure)	[Resin] Barium silicate glass, Methacrylic esters (Bis-MPEPP, others), and others. [Bonding] Methacrylic esters (4-META, others), acetone, water, and others (Brush: aromatic amines, aromatic sulfinate salts).	1. Mix the bonding with brush for 5 s and apply to the cavity. 2. After 5 s, air-blow 5 to 10 s. 3. Fill the resin core material. 4. Light-cure for 20 s.	**LC2**
MI core LC flow (Light cure)	G-Premio BOND (Light cure)	[Resin] Monomers (Urethane methacrylate, Bis-MEPP), strontium glass. [Bonding] 4-MET, phosphate ester monomers, thiol ester monomers, ethyl methacrylate, acetone, water.	1. Apply the bonding to the cavity using a microbrush. 2. After 10 s, strong air-blow 5 s. 3. Light-cure for 20 s. 4. Fill the resin core material. 5. Light-cure for 20 s.	**MIC**
Filtek Fill & Core Universal shade (Light cure)	Scotchbond Universal Plus Adhesive (Light cure)	[Resin] Methacrylates (Bis-GMA, UDMA, and other methacrylates), inorganic fillers, polymerization catalyst, stabilizers, and colorants. [Bonding] Phosphate ester monomers, methacrylates, polymerization initiators, ethanol, and others.	1. Apply the bonding to the cavity using a microbrush. 2. After 20 s, mild air-blow 5 s. 3. Light-cure for 20 s. 4. Fill the resin core material. 5. Light-cure for 20 s.	**FFC**

**Light curing unit:** Valo LED Curing Light, high-power mode at 1400 mW/cm^2^ (Ultradent, South Jordan, UT, USA).

**Table 2:** Mechanical properties of materials including polymerization depth and shrinkage.

Materials	Volumetric Polymerization Shrinkage (%)	Depth of Cure (mm)
Luminous core LC flow	4.7	5.8
DP-031 Resin core	3.5	5.2
MI core LC flow	4.1	3.4
Filtek Fill & Core	3.6	4.3

Note: Testing conducted by manufacturer.

## Data Availability

This study’s data are available within the article. Detailed data that support the findings of this study are available from the corresponding author, Takashi Hatayama, upon reasonable request.
